# New insight into the virulence and inflammatory response of *Staphylococcus aureus* strains isolated from diabetic foot ulcers

**DOI:** 10.3389/fcimb.2023.1234994

**Published:** 2023-07-28

**Authors:** Yuan Wu, Ti Chen, Yanle Wang, Mao Huang, Yurong Wang, Zhen Luo

**Affiliations:** ^1^ Department of Laboratory Medicine, The Third Xiangya Hospital, Central South University, Changsha, China; ^2^ Department of Laboratory Medicine, Xiangya School of Medicine, Central South University, Changsha, China

**Keywords:** *Staphylococcus aureus*, diabetic foot ulcers, β-toxin, virulence, inflammatory response

## Abstract

*Staphylococcus aureus* strains isolated from diabetic foot ulcers (DFUs) have less virulence, but still cause severe infections. Furthermore, hypovirulent *S. aureus* strains appear to be localized in the deep tissues of diabetic foot osteomyelitis, indicating that the unique environment within DFUs affects the pathogenicity of *S. aureus*. In this study, the cell-free culture medium (CFCM) of *S. aureus* strains isolated from DFUs exhibited higher cytotoxicity to human erythrocytes than those isolated from non-diabetic patients with sepsis or wounds. Among these *S. aureus* strains isolated from DFUs, β-toxin negative strains have less virulence than β-toxin positive strains, but induced a higher expression of inflammatory cytokines. Our study and previous studies have shown that the synergistic effect of phenol-soluble modulin α and β-toxin contributes to the higher hemolytic activity of β-toxin positive strains. However, lysis of human erythrocytes by the CFCM of β-toxin negative strains was greatly inhibited by an autolysin inhibitor, sodium polyanethole sulfonate (SPS). A high level of glucose greatly reduced the hemolytic activity of *S. aureus*, but promoted the expression of interleukin-6 (IL-6) in human neutrophils. However, 5 mM glucose or glucose-6-phosphate (G6P) increased the hemolytic activity of SA118 (a β-toxin negative strain) isolated from DFUs. Additionally, patients with DFUs with growth of *S. aureus* had lower level of serum IL-6 than those with other bacteria, and the CFCM of *S. aureus* strains significantly reduced lipopolysaccharide-induced IL-6 expression in human neutrophils. Therefore, the virulence and inflammatory response of *S. aureus* strains isolated from DFUs are determined by the levels of glucose and its metabolites, which may explain why it is the predominant bacteria isolated from DFUs.

## 1 Introduction

*Staphylococcus aureus* is an important opportunistic pathogen that colonizes various anatomical sites within human body, and causes severe life-threatening diseases, such as skin and soft-tissue infection, endocarditis, pneumonia, osteomyelitis and sepsis (Lowy, 1998). To adapt to different environments, *S. aureus* secretes a variety of virulence factors through a finely tuned complex regulatory network. The virulence of *S. aureus* is associated with numerous toxins and extracellular proteases (Otto, 2014), which cause tissue destruction, damage host cells to acquire nutrients (such as iron) for bacterial growth, and impair the host immune system for bacterial survival (Hongo et al., 2009; Kebaier et al., 2012; Katayama et al., 2013; Nakamura et al., 2013).

Diabetic foot problems are a major cause of hospitalization due to the high prevalence of diabetes, and which are also the most common reason for lower-limb amputations (Lipsky et al., 2020). Diabetic foot ulcers (DFUs) are usually colonized by polymicrobial flora, with *S. aureus* being the predominant bacteria, especially in the mild stage of diabetic foot infections (DFIs) (Lew and Waldvogel, 2004; Heravi et al., 2020). The pathogenesis of DFIs is complex, and the severity of which is a consequence of host- and microbial-related factors. A recent study has shown that the iron acquisition system and pathways involved in cell-surface components associated with adhesion and colonization are the most common virulence factors for the development of *S. aureus*-induced DFIs (Heravi et al., 2020). The most abundant reservoir of iron is heme, which is the cofactor of hemoglobin. To gain access to hemoglobin, *S. aureus* expresses numerous hemolysins to lyse erythrocytes. However, *S. aureus* isolated from DFUs usually has less virulence (Sotto et al., 2008; Tuchscherr et al., 2018). Therefore, some particular virulence factors may help to acquire iron from the host.

The function of superantigens, exfoliative toxins, bi-component leukocidins and the pore-forming toxins, such as α-hemolysin and phenol soluble modulins (PSMs), has been well characterized during DFIs (Cassat et al., 2013; Dunyach-Remy et al., 2016; Loughran et al., 2016). However, some particular features can be observed in diabetic patients (Loughran et al., 2016; Jacquet et al., 2019). For instance, *S. aureus* strains isolated from DFUs exhibit less virulence than those from non-diabetic patients, and low-virulent strains seem to be localized in the deep tissues and bone of diabetic foot osteomyelitis (Tuchscherr et al., 2018). Additionally, the genes encoding clp proteases, associated with the misfolded protein response, are upregulated in *S. aureus* isolated from infected diabetic mice. These genes are partially regulated by glucose and affect the hemolytic activity of *S. aureus* (Jacquet et al., 2019). Therefore, extracellular proteases secreted by *S. aureus* from DFUs may play an important role in the development of DFIs.

Diabetes is a complex metabolic disorder that affects serum glucose as well as other sugars, such as fructose and glucose-6-phosphate (G6P)(Menni et al., 2013 **).** High levels of sugar breed pathogen development, leading to DFIs **(** Seo et al., 2021 **)**. However, excessive glucose intake hinders the production of virulence factors and reduces the severity of *S. aureus* infections (Seidl et al., 2008; Dufresne et al., 2022; Chen et al., 2023). Herein, it was found that *S. aureus* strains isolated from DFUs exhibited higher cytotoxicity to human erythrocytes. Additionally, a negative correlation was observed between the hemolytic activity and the expression of IL-6 in neutrophils. However, the hemolytic activity of *S. aureus* strains was found to be influenced by the levels of glucose and its metabolites, such as G6P.

## 2 Materials and methods

### 2.1 Ethics statement

This study was approved by the Ethics Committee of the Third Xiangya Hospital of Central South University, China (no. 2018-S340). Informed consent from patients was not necessary because the clinical *S. aureus* strains used in the study were obtained as part of standard clinical care. However, informed consent was obtained from all healthy donors providing peripheral blood samples.

### 2.2 Patients and sample collection

Between February 2019 and September 2019, a total of 32 non-duplicate *S. aureus* strains were isolated from inpatients with diabetic foot ulcers (DFUs), which were diagnosed basing on the International Working Group on the Diabetic Foot (IWGDF) Guidelines 2015. At the same period, a total of 32 non-duplicate strains were isolated from non-diabetic inpatient with acute wound infection, and 22 non-duplicate strains were isolated from blood of non-diabetic inpatients with sepsis, derived from several different clinical departments. Specimens of secretion were collected within 48 hours of admission and wounds were irrigated with sterile saline before sampling via a sterilized swab with sufficient pressure over the center of the wound (Rondas et al., 2013). Blood cultures were obtained from non-diabetic inpatients with suspected sepsis when they had a fever. These specimens were then placed into sterile transport containers and sent to the Clinical Microbiology Laboratory at the Third Xiangya Hospital of Central South University within 2 h. All isolates were identified using matrix-assisted laser desorption/ionization time-of-flight mass spectrometry (Bruker Biotyper, Germany).

### 2.3 Bacterial strains and culture conditions

The reference strains *S. aureus* ATCC25923, *S. aureus* ATCC29213, *Staphylococcus epidermidis* RP62A, and *Streptococcus agalactiae* ATCC13813 were kindly provided by Juncai Luo (Tiandiren Biotech, Changsha, China). The *S. aureus* strain RJ-2, a β-toxin positive strain, _△_PSMα and _△_agr strains used in this study were kindly provided by Professor Min Li (Renji Hospital, School of Medicine, Shanghai Jiaotong University, China), which were used in a previous study (Li et al., 2016). Bacteria were routinely cultured at 37.0°C on 5% sheep blood agar plates (Autobio Diagnostics Co., Ltd, China). *S. aureus* strains were then grown in lysogeny broth (LB, Solarbio Life Sciences, China) at 37°C with shaking at 180 rpm, and culture supernatants were collected at 20 h post-inoculation. The hemolytic activity of *S. aureus* affected by glucose was assessed by culturing *S. aureus* strains in 3ml fresh LB medium supplemented with 5-, 10-, 15-, and 20 mM glucose (Sigma-Aldrich) or glucose-6-phosphate (G6P, Sigma-Aldrich) within 12 mL tubes. The cell-free culture supernatants (CFCM) were obtained by filtering through PES filters (0.22 µm pore size; Millipore) and used fresh or stored at -70°C.

### 2.4 Quantitative hemolysis assay

Quantitative hemolysis assays were conducted as described by Ridder et al. (Ridder et al., 2021). Briefly, discarded whole blood from healthy human subjects was washed twice with normal saline and resuspended to a final concentration of 4% (v/v). CFCM of *S. aureus* were added to 4% erythrocyte suspension in equal volumes, to a final volume of 300 µl in 96-well flat-bottom plates, and incubated at 37°C for 90 min. The supernatants were transferred to a new 96-well plate and measured at OD_570_ using a microplate reader. To inhibit hemolytic activity, the CFCM of *S. aureus* was pre-incubated with fetal bovine serum (FBS, Gibco), high density lipoprotein (HDL, Solarbio Life Sciences, China), phenylmethylsulfonyl fluoride (PMSF, Sigma-Aldrich), sodium ethylenediaminetetraacetate (EDTA, Sigma-Aldrich), or sodium polyanethol sulfonate (SPS, Sigma-Aldrich) for 30 min.

### 2.5 Reverse CAMP assays

Sheep blood agar plates were used to analyze synergistic hemolysis by using bacterial cultures. These bacteria were placed close to a streaked culture of *S. agalactiae* ATCC13813. *S. aureus* ATCC25923 was used as a positive control, and ATCC29213 as a negative control. Plates were incubated at 37°C for 24 h before analysis.

### 2.6 Protease assay

Total proteolytic activity was determined using the modified skim milk assay (El-Mowafy et al., 2014). The CFCM of *S. aureus* (0.2 mL) were incubated with 0.8 mL 10% skim milk (Solarbio Life Sciences, China) at 37°C, 1000 rpm for 5 h, and the turbidity of supernatant was measured at OD_570_ using a microplate reader. To inhibit the protease activity, the CFCM of *S. aureus* was pre-incubated with SPS or EDTA for 30 min.

### 2.7 Skin and soft tissue infection model

The mouse skin abscess formation model was performed as described elsewhere (Cho et al., 2012). Briefly, male outbred, immune-competent hairless mice aged between 8 and 10 weeks were used for the model. *S. aureus* strains grown to mid-exponential phase, were washed twice with sterile phosphate buffer saline (PBS) and resuspended in PBS at 1.0×10^9^ CFU/mL. Mice were anesthetized with isoflurane and inoculated with 100μL of *S. aureus* suspension or PBS alone by subcutaneous injection. On the third day, the abscess area was measured, incised, and homogenized in normal saline to quantify the bacterial cells. After completion of the entire procedure, all animals were euthanized. The tissue surrounding the abscess was removed, fixed in a 4% paraformaldehyde neutral buffer solution for 24 h, dehydrated in a graded ethanol series, embedded in paraffin, and sliced into 5µm sections. These sections were then stained with hematoxylin-eosin (H&E).

### 2.8 Analysis of *S. aureus* survival in human blood

Blood samples were collected from healthy donors using 10 mg/mL heparin anticoagulation tubes. *S. aureus* strains were washed twice and resuspended to an OD_570_ of 0.6. Then, 1 mL of heparinized human blood was inoculated with 10 µL of *S. aureus* suspension and incubated at 37°C. After 6 h of incubation, the bacterial cells were appropriately diluted to detect the endpoint numbers of CFUs. The survival rate of the bacteria was determined by comparing it to the initial inoculum.

### 2.9 Isolation of human neutrophils

Peripheral blood was drawn from healthy donors and collected in heparin blood collection tubes. Human neutrophils were isolated from the peripheral blood using a previously described method (Cheung et al., 2015). Briefly, peripheral blood was resuspended in RPMI 1640, layered with Ficoll Hypaque Plus (Sigma-Aldrich), and then centrifuged at 1000 g for 20 min. The supernatant was discarded, and the red blood cell (RBC) pellet was incubated with red blood cell lysis buffer (CWBiotech, China) at a 9-fold volume for 15 min at 37°C to remove erythrocytes. After centrifugation at 1000 g for 15 min, the supernatant was aspirated, and the cell pellet was washed and resuspended in RPMI 1640 to the desired concentration.

### 2.10 Measurement of neutrophils lysis

The lactate dehydrogenase (LDH) release assay was performed as described elsewhere (Cheung et al., 2015). Briefly, the CFCM of *S. aureus* was added to 4.0×10^6^ neutrophils/mL to a total volume of 400 µL in 24-well plates and incubated at 37°C with 5% CO_2_ for 90 min. At the desired times, the samples were centrifuged at 2000 g for 5 min, and the supernatants were collected. The LDH activity in the supernatants was measured using the automatic biochemical analyzer 7600-010 (Hitachi, Japan) according to the manufacturer’s instructions.

### 2.11 Cytokine measurement

Human neutrophils were cultured in RPMI 1640 with 5% FBS. The culture was then supplemented with 200 ng/mL lipopolysaccharide (LPS, Sigma-Aldrich) or/and CFCM of *S. aureus* strains, and further cultured at 37°C and 5% CO_2_. After being cultured for 15 h, the culture supernatants were collected for cytokine measurement. The levels of interleukin-1β (IL-1β) and interleukin-6 (IL-6) were measured using a commercially available ELISA assay kit (Abcam, USA) or an up-conversion chemiluminescence assay (Hotgen Biotech Co., Ltd, Beijing, China), respectively, following the manufacturers’ instructions.

### 2.12 Real-time RT-PCR

The homogenates of *S. aureus*-infected mouse skin tissues were lysed using Trizol (Sigma-Aldrich). Total RNA was extracted using a nucleic acid extraction kit (paramagnetic particle method) (Shanghai BioGerm Medical Technology Co., Ltd) following the manufacturer’s instructions. RNA quality and concentration were evaluated using a NanoDrop 1000 (Thermo Fisher Scientific). DNA was then removed by DNase I and the remaining RNA was reverse transcribed into cDNA using a reverse transcription kit (Thermo Scientific) according to the manufacturer’s instructions. Gene relative expression was determined using the 2^-△△Ct^ method, with the level of transcription relative to the expression of the GAPDH gene. The primers used in this study were reported in a previous study (Hirano et al., 2017).

### 2.13 Data analysis of patients with diabetic foot ulcers

We retrospectively analyzed patients with DFUs who had undergone both microbiological culture and serum IL-6 testing within 48 h of hospitalization between January 1, 2020 and June 13, 2020, at the Third Xiangya Hospital of Central South University, Changsha, China. Our institutional ethics board waived the need for written informed consent for this retrospective study, which evaluated de-identified data without posing any potential risks to patients.

### 2.14 Statistical analysis

GraphPad Prism software version 8.3 was used to perform statistical analysis. Significance levels were calculated using unpaired t-test, paired t-test, one way ANOVA or two-way ANOVA analysis. In cases where continuous variables exhibited skewed distributions, the Mann-Whitney U test was used to evaluate differences between two groups. Statistical significance was defined as *p*<0.05. All error bars represent the standard deviation.

## 3 Results

### 3.1 *S. aureus* strains isolated from DFUs have higher cytotoxicity to human erythrocytes

**Figure 1:**
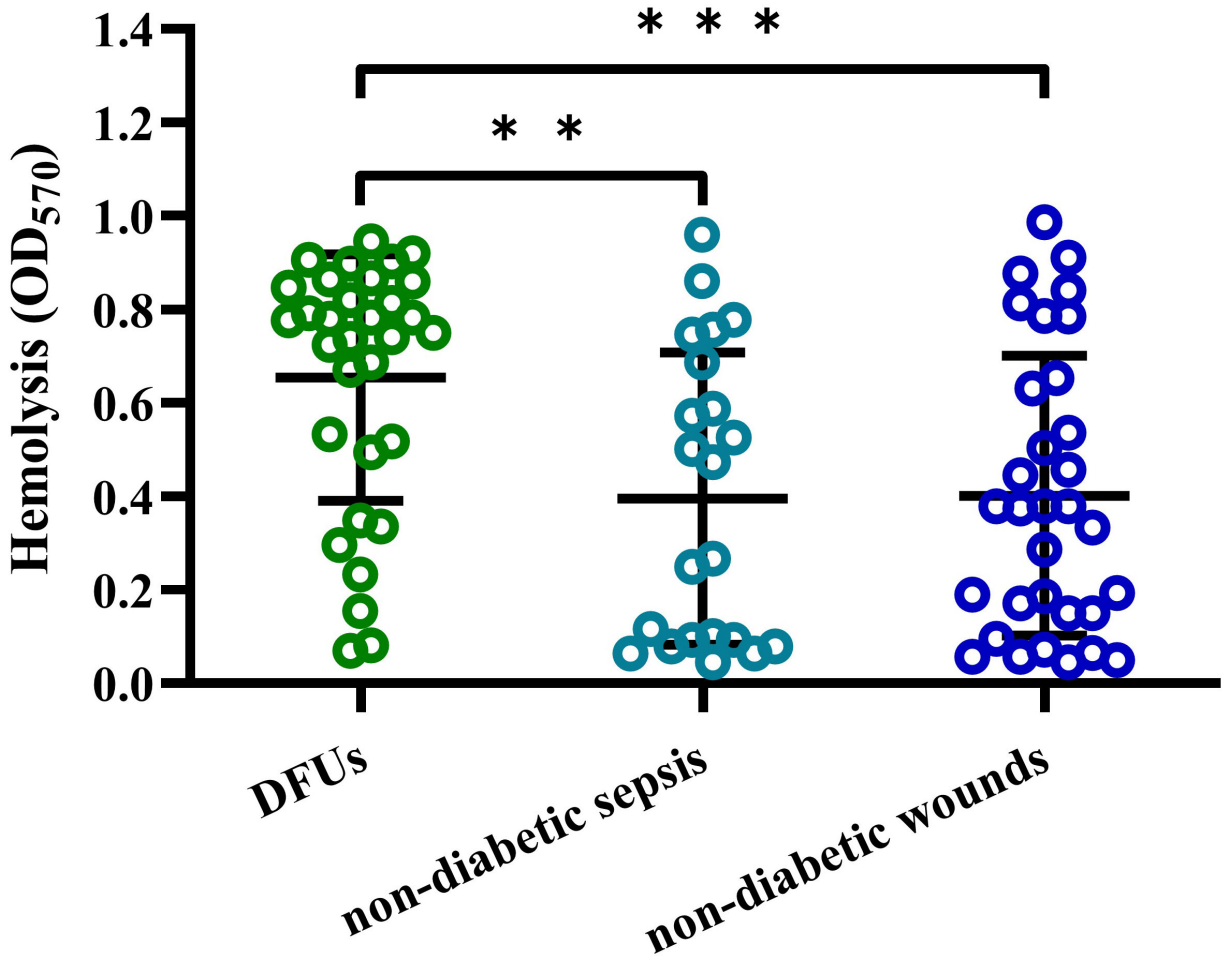
*S. aureus* strain isolated from DFUs had higher hemolytic activity to human erythrocytes. Lysis of human erythrocytes was measured by quantitative hemolysis assays with the CFCM of *S. aureus* strains isolated from DFUs (n=32), non-diabetic sepsis (n=22) and wounds (n=32). ^**^ *p*<0.01, ^***^ *p*<0.001.

To determine the cytotoxicity of *S. aureus* strains isolated from DFUs, we examined their hemolytic activity to human erythrocytes *in vitro*. As shown in **Figure 1**, the hemolytic activity of *S. aureus* isolated from DFUs was higher than those isolated from non-diabetic patients with sepsis or wounds, whereas no significant difference was observed between non-diabetic sepsis and wounds. These data demonstrate that *S. aureus* strains isolated from DFUs have higher hemolytic activity *in vitro*.

### 3.2 β-toxin positive *S. aureus* strains isolated from DFUs have higher virulence

**Figure 2:**
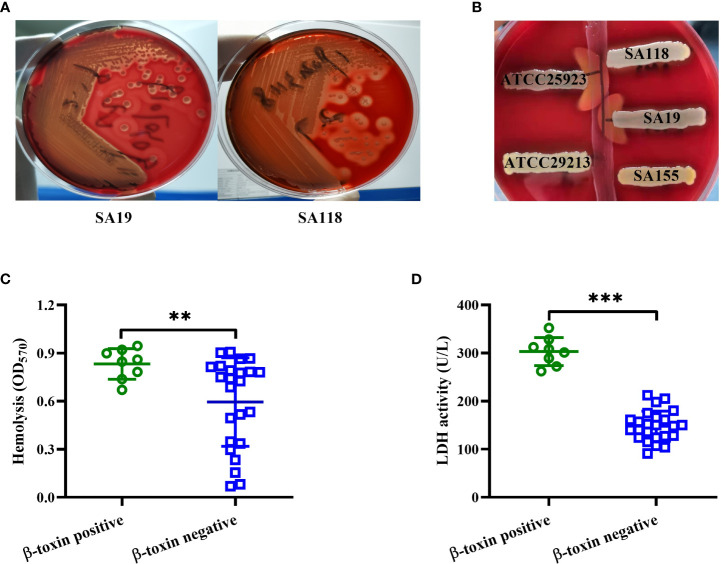
β-toxin positive strains isolated from DFUs had higher virulence *in vitro*. **(A)** Hemolytic phenotype of representative *S. aureus* strains isolated from DFUs (SA19 and SA118 strains) was cultured in 5% sheep blood agar plates. **(B)** Synergistic hemolysis between a strain of *S. agalactiae* ATCC13813 (vertical streak of growth) and five representative *S. aureus* strains (three clinical strains, a positive control ATCC25923 and a negative control ATCC29213). The cytotoxicity of β-toxin positive and negative strains isolated from DFUs to human erythrocytes **(C)** or neutrophils **(D)** was analyzed. ^**^ *p*<0.01, ^***^ *p*<0.001.

*S. aureus* strains isolated from DFUs exhibited complete and incomplete hemolytic phenotypes after culturing strains on 5% sheep blood agar plates (**Figure 2A**). These isolates were further divided into β-toxin positive and negative strains by using the reverse CAMP assay (**Figure 2B**), with 8 out of 32 isolates being β-toxin positive strains. The hemolytic activity of β-toxin positive strains was higher than that of β-toxin negative strains (**Figure 2C**), and higher cytotoxicity of CFCM was observed in β-toxin positive strains when tested against human neutrophils (**Figure 2D**). These data suggest that β-toxin positive strains isolated from DFUs have higher cytotoxicity *in vitro*.

**Figure 3:**
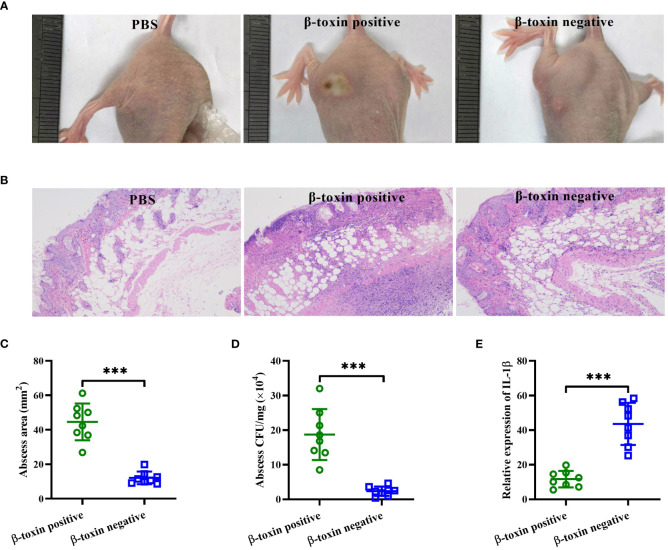
β-toxin positive strains isolated from DFUs had higher virulence *in vivo*. Mice were injected subcutaneously with ~10^7^ CFUs per mouse of β-toxin positive and representative β-toxin negative strains isolated from DFUs (n=8), and control mice received only sterile PBS. **(A)** Representative abscess results were shown on day 2 after infection. **(B)** Representative histological results (H&E stain) were shown on day 2 after infection. **(C)** The abscess areas were measured on day 2 after infection. **(D)** The bacterial load in the infected skin was measured 2 days after infection. **(E)** The expression of IL-1β in the infected tissues was measured by real-time RT-PCR. ^***^ *p*<0.001.

The virulence of β-toxin positive and negative strains isolated from DFUs was further examined using the skin abscess formation model. As shown in **Figures 3A**, **C**, the abscess areas caused by β-toxin positive strains were larger than that caused by β-toxin negative strains. H&E staining results showed that β-toxin positive strains caused greater infiltration of leukocytes and destruction of skin structure than β-toxin negative strains (**Figure 3B**), and the bacterial load was higher in mice infected with β-toxin positive strains (**Figure 3D**). Neutrophils have been shown to be an essential component of the innate immune system involved in the control of *Staphylococcal* infections (De Jong et al., 2019), and neutrophil-derived IL-1β is sufficient for immunity against *S. aureus* skin infection (Miller et al., 2007; Cho et al., 2012). However, the expression of IL-1β mRNA caused by β-toxin positive strains was lower than β-toxin negative strains (**Figure 3E**). These data demonstrate that β-toxin positive strains isolated from DFUs have higher virulence *in vivo*.

### 3.3 Different cytotoxic factors lead to the hemolytic activity of *S. aureus* isolated from DFUs

**Figure 4:**
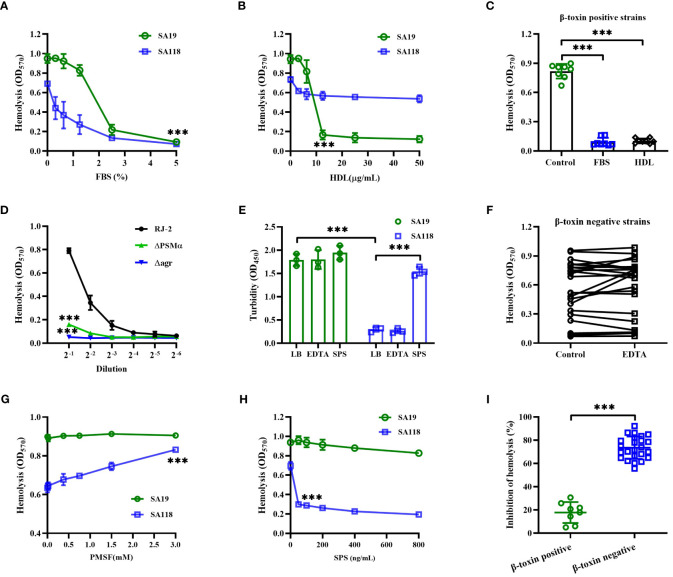
The hemolytic activity of β-toxin positive and negative strains was affected by different cytotoxic factors. The hemolytic activity of SA19 and SA118 strains was analyzed by increasing concentration of FBS **(A)** or HDL **(B)**. **(C)** The inhibition of hemolytic activity by FBS or HDL was analyzed in β-toxin positive strains isolated from DFUs. **(D)** Lysis of human erythrocytes by CFCM of RJ-2, _△_PSMα or _△_agr cultures was analyzed at increasing dilutions. **(E)** The turbidity of skim milk mixed with SA19 and SA118 supernatants was analyzed by pre-incubated with EDTA or SPS. **(F)** The hemolytic activity of β-toxin negative CFCM was analyzed by pre-incubated with EDTA (n=24). The hemolytic activity of SA19 and SA118 supernatants was analyzed by pre-incubated with increasing concentration of PMSF **(G)** or SPS **(H)**. **(I)** The inhibition ratio of hemolytic activity by SPS was analyzed in β-toxin positive and negative strains isolated from DFUs. ^***^ *p*<0.001.

As shown in **Figure 4A**, the hemolytic activity of SA19 (a β-toxin positive strain) and SA118 (a β-toxin negative strain) CFCM were both significantly inhibited by FBS. Previous study has shown that the activity of *Staphylococcal* phenol soluble modulins (PSMs) can be suppressed by serum lipoproteins, such as HDL (Surewaard et al., 2012). The hemolytic activity of SA19 CFCM, but not SA118 strain, was significantly reduced by HDL (**Figure 4B**). The inhibition of hemolytic activity by FBS or HDL was observed in all eight β-toxin positive strains isolated from DFUs (**Figure 4C**). Furthermore, deletion of PSMα or agr gene significantly reduced the hemolytic activity of β-toxin positive strain RJ-2 (**Figure 4D**), and the production of β-toxin was unaffected (data not shown). These data demonstrate that PSMs primarily contribute to the hemolytic activity of β-toxin positive strains.

Next, the skim milk assay was used to determine the proteolytic activity of *S. aureus* strains isolated from DFUs. The OD_570_ value of supernatants showed that the skim milk mixed with SA118 CFCM was less turbidity than that of SA19 strain (**Figure 4E**), suggesting that SA118 strain exhibits higher proteolytic activity. Additionally, the hemolytic activity of β-toxin negative CFCM was unaffected by a metalloprotease inhibitor, EDTA (**Figure 4F**). However, the hemolytic activity of SA118 CFCM was significantly increased by a serine protease inhibitor PMSF, whereas SA19 strain was unaffected (**Figure 4G**). These data suggest that atypical proteases maybe involved in the hemolytic activity of β-toxin negative *S. aureus* strains isolated from DFUs.

Interestingly, SPS, an autolysin inhibitor, greatly reduced the hemolytic activity of SA118 CFCM, but not SA19 strain (**Figure 4H**). The inhibition of hemolytic activity by SPS was 17.82% and 73.54% in β-toxin positive and negative strains, respectively (**Figure 4I**). Additionally, the turbidity of skim milk mixed with SA118 CFCM was significantly increased by SPS, but not by EDTA (**Figure 4E**). Autolysin has been shown to disperse clusters of *S. aureus* (Sugai et al., 1995), and autolysin-deficient cells grow in clusters and have lower proteolytic activity (Takahashi et al., 2002). Herein, most of SA118 cells formed large clusters when grown in presence of SPS, and the hemolytic activity was markedly decreased (**Supplementary Figure 1**). These findings indicate that autolysin may be an important cytotoxic factor of β-toxin negative *S. aureus* strains isolated from DFUs.

### 3.4 Glucose affects the hemolytic activity of *S. aureus* strains isolated from DFUs

**Figure 5:**
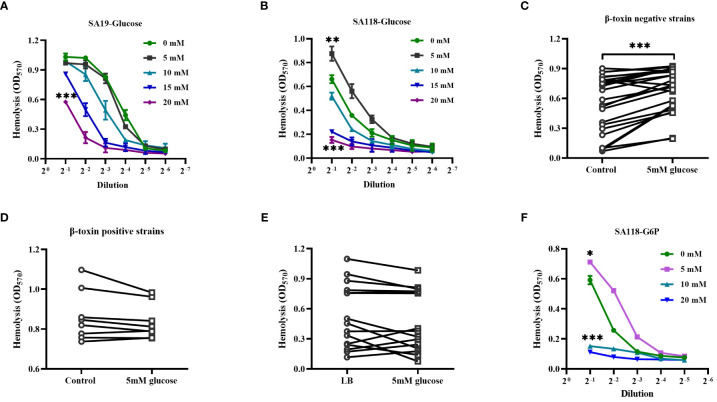
The effect of glucose on the hemolytic activity of β-toxin negative strains isolated from DFUs. Lysis of human erythrocytes by the CFCM of SA19 **(A)** and SA118 **(B)** strains when grown in LB medium with increasing concentration of glucose. Lysis of human erythrocytes was analyzed by the CFCM of β-toxin negative strains **(C)** or β-toxin positive strains **(D)** from DFUs when grown in the LB medium with 5 mM glucose. **(E)** Lysis of human erythrocytes was analyzed by the CFCM of *S. aureus* strains isolated from non-diabetic wounds grown in LB medium with 5 mM glucose (n=16). **(F)** Lysis of human erythrocytes was analyzed by the CFCM of SA118 strain grown in LB medium with increasing concentration of G6P. ^*^ *p*<0.05, ^**^ *p*<0.01, ^***^ *p*<0.001.

Patients with DFUs usually have poorly controlled serum glucose, and it is unclear whether the hemolytic phenotype of *S. aureus* within DFUs is affected by glucose. As shown in **Figures 5A, B**, the hemolytic activity of both SA19 and SA118 CFCM was greatly reduced by the increasing concentrations of glucose. Surprisingly, the hemolytic activity of SA118 CFCM was significantly increased by 5mM glucose (**Figure 5B**). To exclude the possibility of *S. aureus* growth stimulated by glucose, the CFUs were examined. It was found that the growth of SA118 strain was unaffected by 5mM glucose after being cultured for 24 h (data not shown). The increased hemolytic activity by 5mM glucose was observed in most β-toxin negative isolates (**Figure 5C**), but not in the β-toxin positive strains isolated from DFUs (**Figure 5D**). The hemolytic activity of *S. aureus* strains isolated from non-diabetic wounds was also examined in presence of 5mM glucose, which was unaffected (**Figure 5E**). A previous study has shown that diabetes leads to an increase in serum glucose as well as its metabolites like G6P (Seo et al., 2021). The hemolytic activity of SA118 strain was significantly increased by 5mM G6P, but was inhibited by high concentration of G6P (**Figure 5F**). These data indicate that the hemolytic activity of β-toxin negative *S. aureus* strains isolated from DFUs are determined by the level of glucose and its metabolites.

### 3.5 The inflammatory response of *S. aureus* strains isolated from DFUs

**Figure 6:**
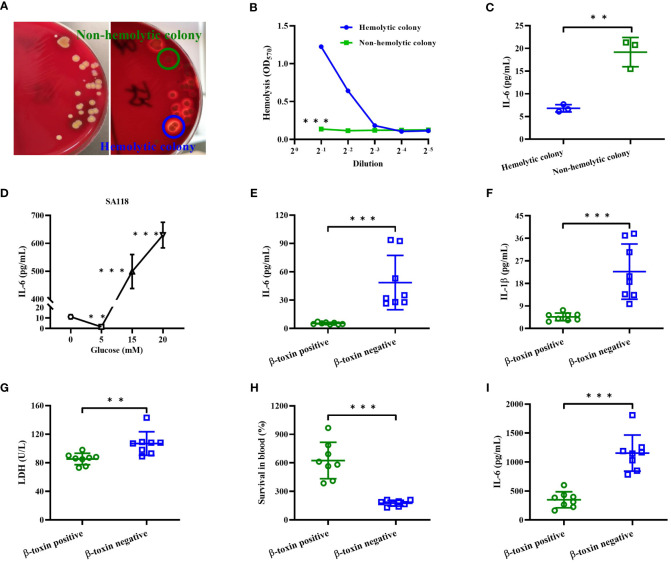
The inflammatory response of *S. aureus* strains isolated from DFUs *in vitro*. **(A)** The hemolytic phenotype of *S. aureus* strains isolated from bone marrow of a patient with diabetic foot osteomyelitis in 5% sheep blood agar plates. **(B)** Lysis of human erythrocytes was analyzed by the CFCM of hemolytic and non-hemolytic colonies. **(C)** The secretion of IL-6 in neutrophils was analyzed by stimulated with the CFCM of hemolytic and non-hemolytic colonies. **(D)** The secretion of IL-6 was analyzed in neutrophils stimulated with the CFCM of SA118 strain when grown in LB medium with increasing concentration of glucose. Human neutrophils were stimulated with CFCM of β-toxin positive or negative strains in presence of FBS for 15 h, and the levels of IL-6 **(E)**, IL-1β **(F)** and LDH activity **(G)** in the culture supernatants were measured. **(H)** The number of CFUs was detected at 0 and 6 h to calculate the rates of survival for β-toxin positive and negative isolates exposed to whole human blood (n=8), and the level of IL-6 in the plasma was measured **(I)**. ^**^ *p*<0.01, ^***^ *p*<0.001.

In a patient with diabetic foot osteomyelitis, *S. aureus* was isolated from both wound and bone marrow. *S. aureus* strain isolated from the wound only showed a hemolytic phenotype after being cultured on 5% sheep blood agar (data not shown), whereas both hemolytic and non-hemolytic colonies were observed in those isolated from bone marrow (**Figure 6A**). Compared to non-hemolytic colony, the CFCM of hemolytic colony showed a higher hemolytic activity to human erythrocytes (**Figure 6B**), but stimulated a lower level of IL-6 in primary human neutrophils (**Figure 6C**). In addition, the level of IL-6 stimulated by the CFCM of SA118 with 15- or 20 mM glucose was higher than those without glucose, but was lower with 5 mM glucose (**Figure 6D**). These data indicate that the levels of glucose affect the hemolytic phenotypic switch and inflammatory response of *S. aureus*.

Next, we examined the pro-inflammatory cytokines released by primary human neutrophils upon stimulation with β-toxin positive or negative CFCM in presence of FBS. Our results showed that β-toxin negative CFCM stimulated higher levels of IL-1β and IL-6 than β-toxin positive strains (**Figures 6E, F**), and a higher LDH activity was observed in the culture supernatants stimulated by β-toxin negative CFCM (**Figure 6G**). Furthermore, we compared the survival rate of *S. aureus* strains by examining the relative viability of β-toxin positive or negative strains in human whole blood. β-toxin negative strains had a lower survival rate compared to β-toxin positive strains (**Figure 6H**), but stimulated a higher level of IL-6 (**Figure 6I**). These data suggest that neutrophil-derived inflammatory cytokines were impaired by β-toxin positive strains isolated from DFUs.

### 3.6 Lower IL-6 expression induced by *S. aureus* strains within DFUs

**Figure 7:**
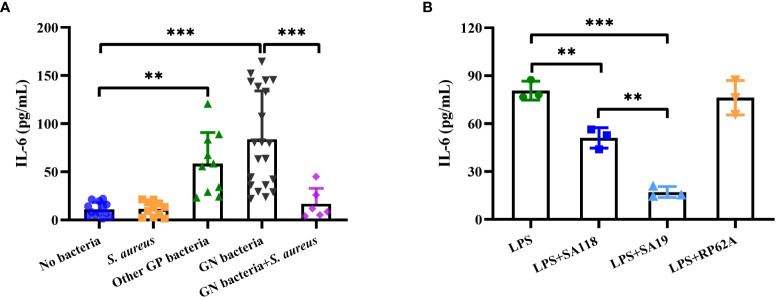
The inflammatory response was impaired by *S. aureus* strains isolated from DFUs. **(A)** The level of serum IL-6 was retrospectively analyzed in patients with DFUs without bacterial growth (n=13) as well as in those with bacterial growth of *S. aureus* (n=11), other Gram-positive bacteria (n=10, included *3 Enterococcus faecalis, 3 Streptococcus dysgalactiae*, 2 *S. agalactiae* and *2 Streptococcus anginosus*), Gram-negative bacteria (n=21, included 8 *Proteus mirabilis*, 4 *Enterobacter aerogenes*, 4 *Morganella morganii*, 3 *Escherichia coli* and *2 Pseudomonas aeruginosa*), or both Gram-negative bacteria (included 3 *E coli*, 2 *E aerogenes* and 1 P*. aeruginosa*) and *S. aureus* (n=7). **(B)** The release of IL-6 was analyzed in neutrophils induced by LPS with the CFCM of SA19, SA118 or *S. epidermidis* RP62A. ^**^ *p*<0.01, ^***^ *p*<0.001.

A previous study has reported that serum IL-6 level seems to be a promising inflammatory marker in the discrimination of infected DFUs (Korkmaz et al., 2018). We retrospectively analyzed the level of serum IL-6 in patients with DFUs. The level of serum IL-6 was higher in those with growth of Gram-negative or other Gram-positive bacteria, whereas no significant difference was observed in the level of serum IL-6 between those with only growth of *S. aureus* and those without bacterial growth (**Figure 7A**). Interestingly, the level of serum IL-6 in those with growth of both Gram-negative bacteria and *S. aureus* was lower than those with growth of Gram-negative bacteria (**Figure 7A**). Next, we examined the release of neutrophil-derived IL-6 induced by LPS with SA19 or SA118 CFCM. As shown in **Figure 7B**, the level of IL-6 induced by LPS was significantly reduced by both SA19 and SA118 CFCM, but not by the CFCM of *S. epidermidis* RP62A. Additionally, the level of IL-6 induced by LPS with SA19 CFCM was lower than that of SA118 strain (**Figure 7B**). These data indicate that *S. aureus* inhibits the inflammatory response and may favor its growth within DFUs.

## 4 Discussion

Several studies have shown that *S. aureus* isolated from DFUs usually exhibit less virulence, but are perfectly adapted to infect deep tissues and bone (Dunyach-Remy et al., 2016; Tuchscherr et al., 2018). Herein, it was found that the *S. aureus* strain isolated from DFUs had higher cytotoxicity to human erythrocytes. *S. aureus* isolated from DFUs were divided into β-toxin positive and negative strains. A previous study has shown that β-toxin positive strains lead to the development of large caseous lesions in pneumonia, and increase the size of pathognomonic vegetations in infective endocarditis (Salgado-Pabon et al., 2014). Additionally, β-toxin promotes *S. aureus* skin colonization by damaging keratinocytes, and the colonization efficiency of β-toxin positive strains on mouse ears is more than 50-fold greater than that of β-toxin negative strains (Katayama et al., 2013). Herein, β-toxin positive strains isolated from DFUs showed higher virulence than β-toxin negative strains, but induced a lower inflammatory response. Therefore, β-toxin positive strains appear to colonize in DFUs more easily and cause more severe infections compared to β-toxin negative strains.

β-toxin is a sphingomyelinase encoded in most *S. aureus* strains, but the production of β-toxin was uncommon in human *S. aureus* isolates due to the widely distributed *hlb* gene inactivating bacteriophage (Van Wamel et al., 2006). Inactivation of β-toxin gene strongly reduced the hemolytic activity of *S. aureus* to human erythrocytes (Salgado-Pabon et al., 2014; Jung et al., 2017). Herein, only 8 out of 32 *S. aureus* isolates from DFUs produced β-toxin, and β-toxin positive strains had higher virulence than β-toxin negative strains. The cytotoxicity of β-toxin is not as efficient as other toxins, but enhances the activity of PSMs (Cheung et al., 2012). PSMs have been identified as a key contributor to infection with *S. aureus*, and which are regulated by the accessory gene regulator (*agr*) quorum-sensing system (Janzon and Arvidson, 1990; Kretschmer et al., 2012). PSMs strongly affect the capacity of the *S. aureus* to lyse many types of eukaryotic cells, including human erythrocytes and neutrophils (Li et al., 2016). We observed that deletion of PSMα or *agr* gene significantly reduced the hemolytic activity of β-toxin positive strain RJ-2, but the production of β-toxin was not affected. In addition, the hemolytic activity of β-toxin positive CFCM was inhibited by FBS due to the inactivation of PSMs activity by lipoprotein particles (Surewaard et al., 2012). Therefore, the enhanced hemolytic activity of β-toxin positive strains isolated from DFUs is due to the synergistic hemolysis of β-toxin and PSMs.

PSMs have been shown to be the main virulence factors of *S. aureus* responsible for inflammatory mediator induction in human keratinocytes (Damour et al., 2021), and which are critical for the early leucocyte influx to the site of infected skin (Nguyen et al., 2022). Herein, more infiltration of leucocytes was observed in the skin infected by β-toxin positive strains than β-toxin negative strains, but had impaired local inflammatory response and increased the bacteria load. There is a suppressed transcriptional signature that is conserved across *S. aureus* lineages, leading to a decrease production of cytokines and chemokines (Zwack et al., 2022). Herein, patients with DFUs with growth of *S. aureus* usually had lower level of serum IL-6 than those with growth of Gram-negative or other Gram-positive bacteria. Neutrophils are an essential component of the innate immune system involved in control of *staphylococcal* infections (De Jong et al., 2019). We observed that the expression of IL-6 in neutrophils induced by LPS was greatly decreased by SA19 or SA118 CFCM, but was not affected by *S. epidermidis* RP62A. In addition, the hemolytic activity of SA118 strain was decreased by high glucose, but induced higher expression of IL-6 in human neutrophils. These data indicate virulence factors secreted by *S. aureus* leads to a decrease production of inflammatory cytokines. It is likely that the lack of these virulence factors explains why we do not observe the same suppression signature induced by *S. epidermidis* RP62A.

*S. aureus* secretes a range of extracellular proteases, some of which are known virulence factors (Michel et al., 2006; Pietrocola et al., 2017). The majority of *S. aureus* isolated from DFUs were β-toxin negative strains. Their hemolytic activity was significantly inhibited by FBS, which contained a large amount of protease inhibitors, but not by HDL. In addition, the CFCM of SA118 strain exhibited higher proteolytic activity. These findings indicate that the extracellular proteases contribute to the higher hemolytic activity of β-toxin negative strains. The hemolytic activity and proteolytic activity of β-toxin negative strains were greatly inhibited by SPS. In addition, PMSF has been shown to increase the release of extracellular autolysin activity (Fournier and Hooper, 2000), which significantly increased the hemolytic activity of SA118 strain. Deletion of *Alt* gene has been shown to reduce the extracellular bacteriolytic activity of BH1CC strain (Houston et al., 2011). These data indicate that autolysin may contribute to the hemolytic activity of β-toxin negative *S. aureus* strains isolated from DFUs. SPS has been shown to suppress the activity of different autolytic wall systems in normally growing staphylococci, but there is no direct evidence to support that SPS directly interacts with the autolytic wall enzymes (Wecke et al., 1986). Recent studies have shown that autolysin creates depots for LukAB in the cell envelope by breaking down the peptidoglycan strands, which in turn indirectly reduces the cytotoxicity of *S. aureus* against human neutrophils (Zheng et al., 2021; Zheng et al., 2022). Therefore, autolysin may be an important cytotoxic factor for β-toxin negative *S. aureus* from DFUs, but the exact mechanism needs further investigation.

The virulence of *S. aureus* is influenced by their intrinsic virulence profile and the environmental conditions, and the environmental stress usually induces hemolytic phenotypic switch (Tran et al., 2019). Herein, the hemolytic phenotypic switch was observed in a patient with diabetic foot osteomyelitis. Patients with DFUs are usually accompanied by abnormal level of serum glucose, and high level of glucose has been shown to reduce the bacteriolytic activity of BH1CC strain and the expression of virulence factors (Houston et al., 2011; Dufresne et al., 2022). We observed that high levels of glucose greatly reduced the hemolytic activity of *S. aureus* strains isolated from DFUs. A previous study has reported that the level of glucose in wound fluid ranges from 0.6 to 5.9 mM (Trengove et al., 1996). It was found that 5 mM glucose significantly increased the hemolytic activity of β-toxin negative strains isolated from DFUs, so *S. aureus* isolated from the superficial wounds in patients with DFUs usually had higher hemolytic activity. The increased hemolytic activity by 5mM glucose is a common phenomenon in the β-toxin negative strains isolated from DFUs, which was not observed in the β-toxin positive strains and those strains isolated from non-diabetic wounds, suggesting that *S. aureus* strain forms unique virulence properties within DFUs. Except for glucose, G6P is also an important metabolic signal for *S. aureus* and enhances the virulence in diabetes (Seo et al., 2021). The hemolytic activity of SA118 strain was increased by 5mM G6P. Therefore, the special environment within DFUs maintains the growth and virulence of *S. aureus.*

## Data availability statement

The raw data supporting the conclusions of this article will be made available by the authors, without undue reservation.

## Ethics statement

This study was approved by the Ethics Committee of the Third Xiangya Hospital of Central South University, China.

## Author contributions

YW, TC and ZL designed the study, YW, TC, YLW, MH and YRW conducted the experiments, YW, TC and ZL analyzed the results. YW, TC and ZL wrote the manuscript, and YLW, MH and YRW reviewed the manuscript. All authors contributed to the article and approved the submitted version.

## Supplementary material

Supplementary Figure 1 Representative morphology **(A)** and hemolytic activity **(B)** of SA118 strain was analyzed when grew in LB medium in presence of SPS. ****p*<0.001.
