# Supplementary figures and images for: New insight into the virulence and inflammatory response of Staphylococcus aureus strains isolated from diabetic foot ulcers
Source: Front Cell Infect Microbiol. 2023 Jul 28;13:1234994. doi: 10.3389/fcimb.2023.1234994 (PMC10416727; doi:10.3389/fcimb.2023.1234994)

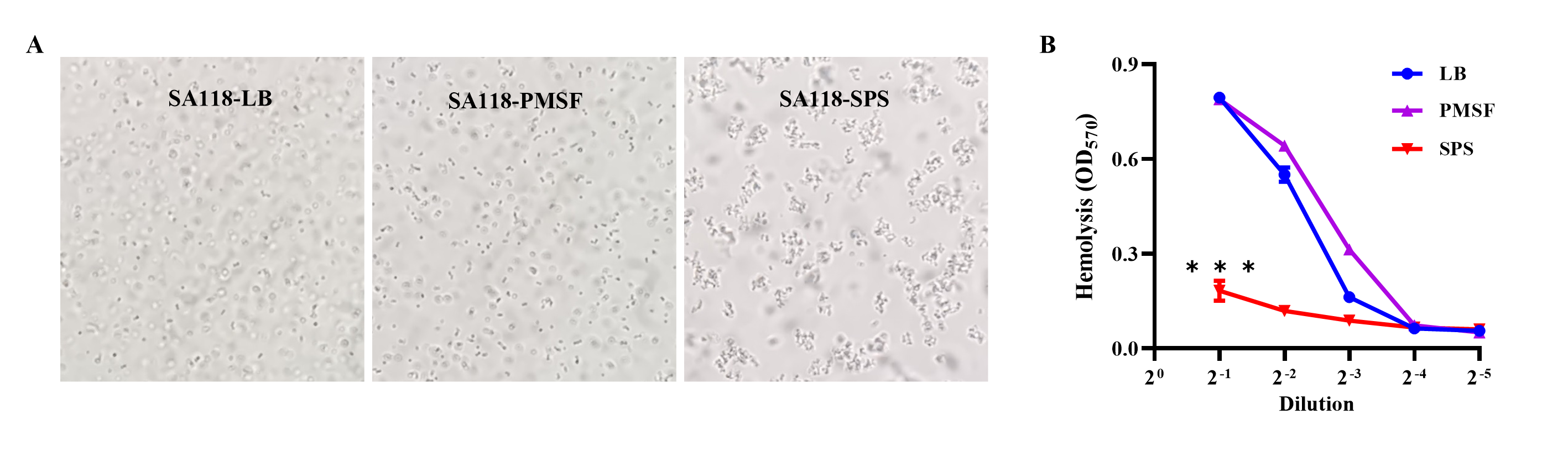

Supplement: Supplementary Figure 1 — Representative morphology (A) and hemolytic activity (B) of SA118 strain was analyzed when grew in LB medium in presence of SPS. ***p<0.001. [file Image_1.tif]
